# HGF-Transgenic MSCs Can Improve the Effects of Tissue Self-Repair in a Rabbit Model of Traumatic Osteonecrosis of the Femoral Head

**DOI:** 10.1371/journal.pone.0037503

**Published:** 2012-05-21

**Authors:** Qian Wen, Dan Jin, Chao-Ying Zhou, Ming-Qian Zhou, Wei Luo, Li Ma

**Affiliations:** 1 Institute of Molecular Immunology, Southern Medical University, Guangzhou, People's Republic of China; 2 Department of Orthopaedics and Traumatology, Nanfang Hospital, Southern Medical University, Guangzhou, People's Republic of China; Van Andel Institute, United States of America

## Abstract

**Background:**

Osteonecrosis of the femoral head (ONFH) is generally characterized as an irreversible disease and tends to cause permanent disability. Therefore, understanding the pathogenesis and molecular mechanisms of ONFH and developing effective therapeutic methods is critical for slowing the progress of the disease.

**Methodology/Principal Findings:**

In this study, an experimental rabbit model of early stage traumatic ONFH was established, validated, and used for an evaluation of therapy. Computed tomography (CT) and magnetic resonance (MR) imaging confirmed that this model represents clinical Association Research Circulation Osseous (ARCO) phase I or II ONFH, which was also confirmed by the presence of significant tissue damage in osseous tissue and vasculature. Pathological examination detected obvious self-repair of bone tissue up to 2 weeks after trauma, as indicated by revascularization (marked by CD105) and expression of collagen type I (Col I), osteocalcin, and proliferating cell nuclear antigen. Transplantation of hepatocyte growth factor (HGF)-transgenic mesenchymal stem cells (MSCs) 1 week after trauma promoted recovery from ONFH, as evidenced by a reversed pattern of Col I expression compared with animals receiving no therapeutic treatment, as well as increased expression of vascular endothelial growth factor.

**Conclusions/Significance:**

These results indicate that the transplantation of HGF-transgenic MSCs is a promising method for the treatment for ONFH and suggest that appropriate interference therapy during the tissue self-repair stage contributes to the positive outcomes. This study also provides a model for the further study of the ONFH etiology and therapeutic interventions.

## Introduction

Traumatic osteonecrosis of the femoral head (ONFH) is a progressive pathological process primarily caused by interrupted blood circulation in the femoral head that leads to apoptosis of endothelial cells [1] and hemopoietic/osseous tissue necrosis [2]. The pathology then progresses through several stages [2]. First, there is a repair stage, which generally begins shortly after cell death and is accompanied by the proliferation of undifferentiated mesenchymal cells as well as capillary buds containing endothelial cells around the fracture. Next, there is a remodeling stage, which is established by the concomitant appearance of osteoclasts (OCs), mesenchymal cells, and capillaries; this stage involves repeated trabecular microfractures due to the action of mechanical factors on the femoral head, which is comprised of various materials of diverse elasticities. Finally, there is a joint impairment stage, during which impairment originates from the fracture at the junction between dead and live bone due to their differing elastic moduli and compliance; this impairment usually occurs after a substantial delay, approximately 2 years after the femoral neck fracture. Without timely, effective treatment, the patient may be left with a permanent disability. Clinical signs typically include hip pain, followed by progressive lameness that results in the partial or complete inability to bear weight on the affected limb.

Many challenges remain in the treatment of ONFH. Firstly, effective therapeutic methods are lacking. This lack may be due to insufficient understanding of the pathogenesis of osteonecrosis (e.g. the posttraumatic molecular events). Secondly, regarding treatment selection, it is unclear whether surgery is the best therapy for ONFH. Other treatment options, such as an auxiliary means of activating the patient's healing systems, may be better. Furthermore, because there is usually no need to change the articulus of patients with artificial one during the early stage of traumatic ONFH, it is difficult to acquire tissue samples for diagnosis. Molecular level events at the lesion site have not been well addressed and there is insufficient information available to improve the timing and efficacy of treatment. An animal model of early ONFH that truly simulates the clinical situation would provide a platform for the study of the pathogenesis and molecular mechanisms of the disease, and would contribute to the development of an optimal treatment regimen.

To establish a traumatic ONFH model, several methods for blocking the blood supply to the femoral head have been reported; these involve tightening the femoral blood vessels, circulation of liquid nitrogen around the femoral head, and dislocation of the hip and detachment of the soft tissue [3], [4], [5], [6], [7], [8], [9]. Nishino *et al*. compared several methods for establishing a traumatic ONFH model using hematoxylin and eosin (H&E) staining; they found that a combination of dislocation and ligation of femoral vessels, but not either one alone, led to empty lacunae, significant necrosis, and vigorous appositional bone formation in 80% of animals after 2 weeks [10]. Their results suggested that none of the methods ensured that all animals would exhibit the typical symptoms of ONFH, and that a relatively severe method is needed. In the present study, the femoral neck was broken completely, including the round ligament, severely disrupting blood circulation in the femoral head. This method is relatively more acute than other methods, has a higher success rate in a shorter time, and is convenient for further studies. In addition, the model can be treated through fixation with a small splint to assess the efficacy of therapeutic regimens.

Clinically, the lack of effective therapy for ONFH has become an issue that needs to be overcome. Several methods for the treatment of early stage traumatic ONFH have been attempted, including drug therapy, surgical core decompression, vascularized bone grafting, and osteotomy. However, none of these has been effective in preventing the disease. Femoral heads usually collapse in the late stage of ONFH and the only feasible treatment is arthroplasty. However, the failure rate after 5 years is 10%–50%, and the best artificial femoral heads only last for 10–15 years. Thus, patients often need two or three replacements, entailing pain and a further economic burden. Thus finding early treatment that could save the femoral head would represent an important advance.

Mesenchymal stem cells (MSCs) are pluripotent and can differentiate into several lineages of cells, including osteocytic, chondrocytic, and adipocytic cells. They are present in the adult bone marrow and have shown potency in the treatment of ischemic diseases, such as myocardial infarction [11], [12] and ONFH 13,14. In our previous work, we treated early stage hormone-induced ONFH with the transplantation of MSCs infected with replication-deficiency adenoviral vectors carrying the hepatocyte growth factor (HGF) gene, with relatively satisfactory therapeutic efficacy [15]. In that study, after MSC transplantation, changes in HGF protein levels in the region of the disease correlated with the sequential activation or inhibition of the p-ERK1/2 or p-Akt pathways. These sequential changes contribute greatly to the function of MSCs in the recovery of injured tissue [16].

In the present study, a rabbit model was established to study molecular events during the pathogenesis of traumatic ONFH and to assess the efficacy of transplantation of HGF-transgenic MSCs. The model was validated by radiological and histopathological examinations, which suggested the occurrence of tissue self-repair. Coordinated with self-repair, we found HGF-transgenic MSC transplantation to be efficacious in the treatment of the traumatic ONFH model.

## Materials and Methods

### Animals

Healthy, male New Zealand rabbits aged 3 months and weighing between 2.8–3.3 kg were provided by the Experimental Animal Centre of Nanfang Hospital, Southern Medical University (Guangzhou, China), and were maintained under specific pathogen-free conditions. All animals received humane care in compliance with the Guide for the Care and Use of Laboratory Animals, published by the US National Institutes of Health (Publication No. 85-23, revised 1996). The experiment protocol was approved by the Animal Ethics Committee at Southern Medical University.

### Operation performance

Five rabbits were set aside as intact controls, while 40 rabbits underwent traumatic surgery and were evaluated using radiological and histopathological examinations at different time-points up to 3 weeks as indicated in Table 1. Animals were anaesthetized with an intravenous injection of 30 mg·kg^−1^ body weight 30% pentobarbitone sodium (Sigma, MO, USA). A posterolateral incision was made in the left hip under aseptic conditions, and a 3 cm incision was made in the joint capsule to expose the femoral head. All soft tissue attachments, including the annular ligament, were detached, and the femoral neck was severed at the base. An unabsorbable suture was used to secure the separated femoral head into the acetabulum, and the wound was copiously irrigated with penicillin before being closed in layers. Animals received 2×10^5^ U of penicillin intramuscularly as prophylaxis. During the post-operative period, all animals were free to move, and recovery from anaesthesia without post-operative infection was observed. However, difficulty in jumping was observed.

**Table 1:** Animal observation groups.

Observation group	Total number of hips (animals) per group	Number of hips (animals) in analysis groups	
		Radiology	Histology (H&E/IHC)
Normal	10(5)	4(2)	6(3)
3 days post operation	10(10)	4(4)	6(6)
1 week post operation	10(10)	4(4)	6(6)
2 week post operation	10(10)	4(4)	6(6)
3 week post operation	10(10)	4(4)	6(6)

### CT examinations

Radiographs were obtained at various intervals post-operatively using a Lightspeed 16 spiral CT scanner (GE Company, New York, USA). After injection of 1.5 mL veterinary Sumianxin II, a compound anesthetic preparation consisting of haloperidol, Baoding Ning, dihydrocodeine Eto'o, etc., into the gluteal muscle for anesthesia, animals were fixed in the supine position, with hip joints positioned as symmetrically and laterally as possible. Transect scanning of the entire hip joint was performed at 100 kV and 220 mA. The screw pitch was 0.625∶1, the combination detector was 16×0.625 mm, the bed speed was 5.62 mm·r-1, the reconstruction layer was 1.25 mm thick, and the diameter of the field-of-view (FOV) was 9.6 cm.

### MRI examinations

A Magnetom Vision PLUS 1.5 T superconducting MRI machine (Siemens, Germany) was used. After being anaesthetized in the same session for the CT, animals were placed supine on a bed and the centre of an orthogonal head coil was located on the hip joint. Sequences included T1-weighted spin-echo (TR = 440.0, TE = 6.9), fat suppressed T2-weighted fast spin-echo (TR = 4000.0, TE = 87.0). Images were collected twice in the coronal position. The layer thickness was 3 mm and the FOV was 100×100 mm. Due to truncation of the femoral neck, no contrast agent could be administered into the femoral head to perform post-contrast MR imaging.

### Histopathological examinations

Femoral head specimens were fixed in 4% buffered paraformaldehyde, decalcified in buffered 10% ethylenediamine tetraacetic acid disodium salt (Na_2_-EDTA) for more than 20 d. After being dehydrated stepwise and turned transparent by dimethylbenzene, the samples were embedded in wax and sectioned into coronal planes (4 µm). Sections were stained with H&E to observe histological changes after injury. Image-Pro Plus 6.0 software (IPP 6.0, Media Cybernetics, MD, USA) was used to quantify the number of empty lacunae and haematopoietic cell nuclei observed at 100× magnification in ten randomly selected fields per section. The width and area of trabeculae and the area of grey scale in the trabeculae mainly under cartilage were also calculated.

Immunohistochemistry (IHC) was performed using antibodies specific for: collagen type I (Col I) (I-8H5; Calbiochem, Darmstadt, Germany), osteocalcin (OCN) (OCG4; Abcam plc., Cambridge, UK), von willebrand factor (vWF) (F8/86), phosphorylated-ERK1/2 (p-ERK1/2) (E-4) (Santa Cruz Biotechnology, CA, USA), CD105 (SN6h), proliferating cell nuclear antigen (PCNA) (PC10) (Invitrogen co. Ltd., CA, USA), HGF, Vascular endothelial growth factor (VEGF) (BOSTER Bioengineering Co. Ltd., Wuhan, China) and phosphorylated-Akt (p-Akt) (D9E; Cell Signaling Technology, MA, USA). Briefly, sections were incubated with primary antibodies at 4°C overnight, washed 3 times, then incubated with biotinylated secondary IgGs (Maxim Bio., Fujian, China). Sections were washed again, then developed with 3,3′-diaminobenzidine (DAB) (Zhongshan Goldenbridge Biotechnology, Beijing, China). Sections incubated without primary antibodies were used as a negative control. All specimens were consistently maintained in liquid to prevent edge effects. Each section was imaged at 200× magnification, and IPP 6.0 was used for quantitation using a single blind method. In bone marrow cavities of ten randomly selected fields, staining was quantitated based on integrated optical density (IOD). The corresponding cavity area was also measured, and the expression ratio was calculated by dividing IOD by the cavity area. For Col I, the expression ratio for both bone marrow and trabeculae were calculated.

### Isolation and culture of MSCs and Differentiation assays

Allogeneic MSCs were achieved and adenoviral vectors (Ad-vectors) carrying or not HGF gene (Ad-HGF) were prepared as described before with some modification [15]. Briefly, bone marrow was aspirated from the bilateral posterior superior iliac spine of rabbits and washed with Dulbecco's modified Eagle's medium-low glucose (Hyclone Ltd, Logan, UT, USA) containing 10% fetal bovine serum (FBS; Hyclone) and 100 U/ml penicillin, 100 mg/ml streptomycin and 2 mM L-glutamine (Invitrogen). The adherent cells were cultured for two generations and then infected with Ad-vectors or Ad-HGF at a multiplicity of infection (MOI) of 300 before transplantation. For identifying the pluripotency of MSCs, cells were induced to differentiate into osteoblasts, chondroblasts and adipocytes respectively according to the methods described before [16].

### Treatment of traumatic ONFH with transplantation of allogeneic MSCs

For treatment, traumatic ONFH was induced in 48 rabbits. One week later, 10^6^ cells in 100 µL culture medium without FBS were transplanted into the necrotic femoral head exposed and closed in the same way as model establishment. Three animals were left untreated as model controls (ONFH). Fifteen animals were transplanted with HGF-transgenic MSCs (ONFH+MSC+HGF). Another 15 rabbits received adenoviral vector-infected MSCs as vector controls (ONFH+MSC+vector). The remaining 15 animals were transplanted with uninfected MSCs as treatment controls (ONFH+MSC). Then the leg was fixed with a small splint and the animals were carefully fed. Four weeks later, the treatment efficacy was assessed through pathological examination. During the experiment, no animal unintentionally died. At 2 d, 2 weeks, and 4 weeks after transplantation, euthanization were performed by air injection through ear vein to obtain the femoral head samples. Paraffin samples and sections were sequentially prepared for H&E staining and IHC assays.

### Statistical analysis

SPSS statistical software (version 16.0; SPSS, Chicago, IL, USA) was used. Data are expressed as the mean ± standard deviation of the mean. One-way ANOVA was applied to determine statistical significance. Least-significant differences, or Dunnett's T3 tests, were performed for post-hoc multiple comparisons. A *P*-value less than 0.05 was considered statistically significant.

## Results

### Radiological inspection of traumatic ONFH model

To verify whether the model represents the typical clinical Association Research Circulation Osseous (ARCO) phase I or II of ONFH, we conducted CT and MR imaging.

**Figure 1:**
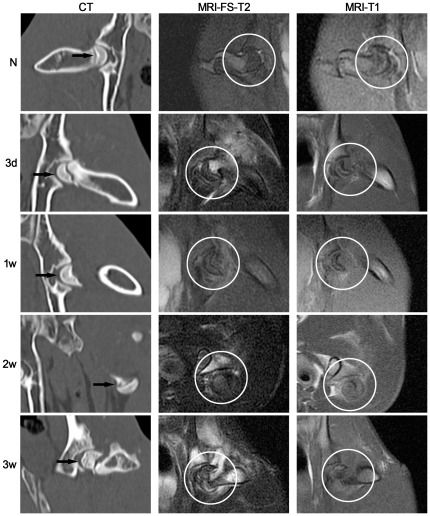
Conventional CT and MR examination of the traumatic ONFH rabbit model established in this study. Left column: Conventional CT examination (coronal reconstruction). With progression of the disease, the density of cancellous bone became more heterogeneous and eventually lost its symmetry. Arrows indicate the femoral head. Middle column: MR-FS T2-weighted imaging (coronal plane) detected a signal that became increasingly uneven for up to 2 weeks after trauma. A signal with high intensity was observed at the fracture line, suggesting edema was present in the bone marrow. Right column: MR T1-weighted imaging (coronal plane) detected a loss of normal signal within the femoral head. Circles indicate the femoral head. N: normal group; 3 d, 1 w, 2 w, 3 w: 3 days, 1 week, 2 weeks, 3 weeks post-trauma, respectively.

CT examinations of normal rabbits showed symmetrical femoral heads, clear and distinct joint surface and epiphyseal line, and a smooth and intact cortex. In contrast, femoral heads that had undergone a severance of the annular ligament and the femoral neck to severely damage blood circulation, exhibited an increasingly heterogeneous density of cancellous bone, a gradual loss of symmetry, thinning of the bone cortex, and the epiphyseal line was blurred (Figure 1, left column).

MR imaging features of the normal femur include uniformly high signal intensities compared with muscle. Pathological changes are characterized by inhomogeneous intermediate to high signal intensity on MR FS T2-weighted images in which the subcortical high intensity fat signal is suppressed and is reversed on T1-weighted images within the femur compared with muscle. On T2-weighted images, originally uniform signal intensity within the operative coxofemoral joint began to increase unevenly 2 weeks after trauma, especially within the fracture line, suggesting the presence of marrow edema (Figure 1, middle column). Similarly, on T1-weighted images, the injured femoral head had a smooth surface that became inhomogeneous 2 weeks after trauma, except for a band-like area of low signal intensity in the distal stump marrow of the femoral neck, which was seen as early as 3 days after trauma (Figure 1, right column). Similar changes were observed at up to 3 weeks on both sequences in all traumatized rabbits, more than two-thirds of which also lacked signal intensity locally in the subchondral bone. In comparison, the unaffected femurs of these rabbits maintained a uniform signal. Overall, prominent changes in signals associated with cancellous bone were detected by week 3, consistent with the report of Nishino *et al* [10] and the imaging features of ONFH in ARCO phase I and II 17,18,19.

### Pathological examination validated the ONFH model

**Figure 2:**
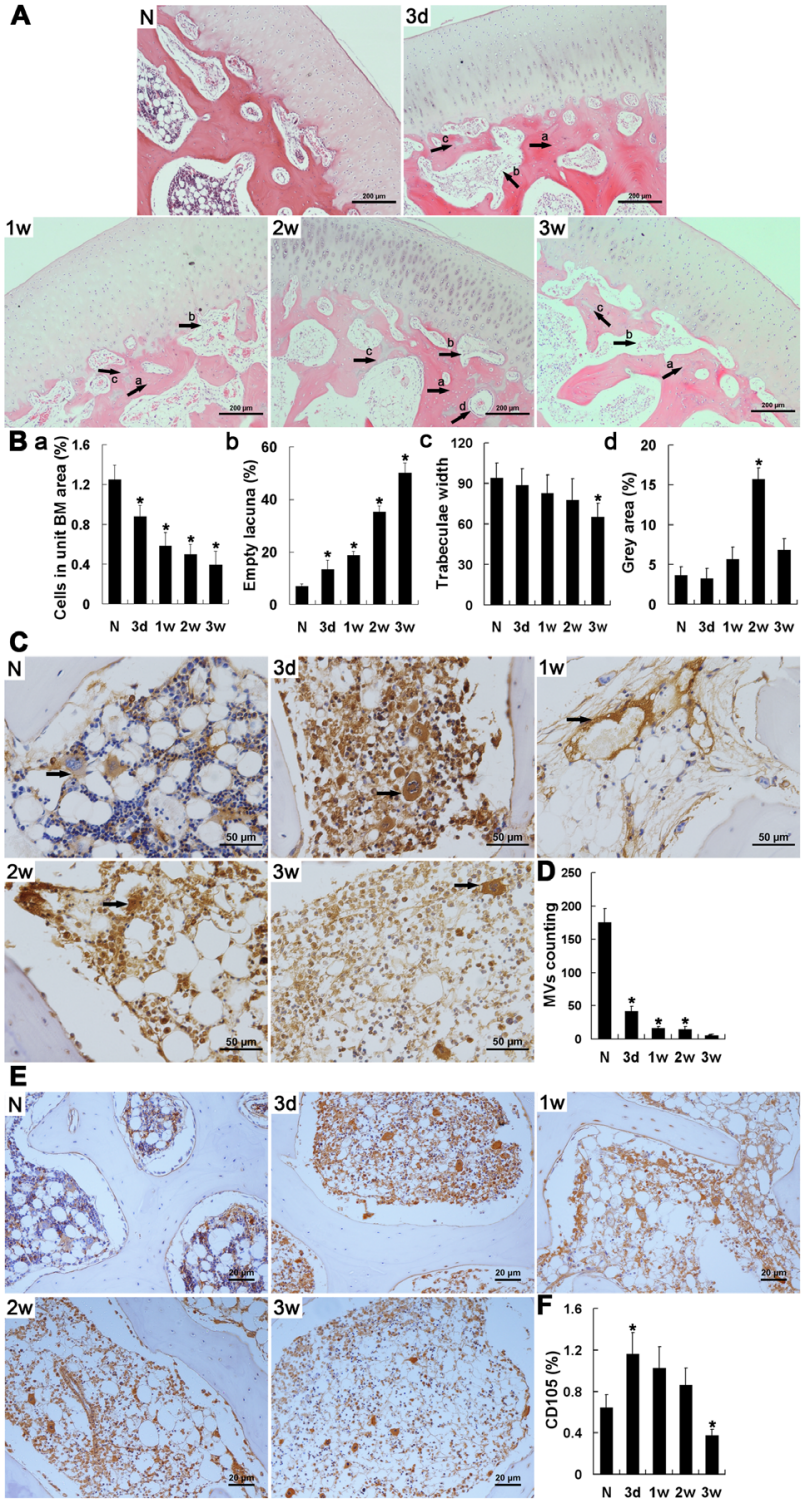
Histopathological images of the traumatic ONFH rabbit model, and immunohistochemical staining and semi-quantitative analysis of vWF and CD105. (A) Representative images of a section of femoral head including cartilage stained with H&E. Scale bar = 200 µm. In the injured femoral head, the number of empty lacunae increased and hematopoietic tissue diminished significantly, but cartilage did not exhibit any obvious pathological changes. Immature fibrotic tissue and appositional bone formation were observed under the cartilage from 3 days after trauma, followed by an increase in the number of OBs 2 weeks later. a. empty lacuna; b. immature fibrotic tissue; c. appositional bone formation; d. OBs. (B) Bar graphs represent the ratio of bone marrow cells to the area of bone marrow (a), the ratio of empty lacunae to the area of trabeculae (b), thinning trabeculae (c) and grey scale (d), respectively. (C) Immunohistochemical assays of vWF expression. Scale bar = 50 µm. (D) Blood vessels were counted according to positive staining of vWF in combination with appropriate vessel structure. Following trauma, the structure of the blood vessels became increasingly compromised and the number of blood vessels decreased. Arrows = microvessels or arteries. (E) Immunohistochemical assays of vWF expression. Scale bar = 20 µm. (F) Bar graphs represent the expression density of CD105 as unit area of bone marrow or unit area of bone trabeculae. The expression of CD105 until 3 weeks after trauma suggested the presence of revascularization in the injured femoral head. N: normal group; 3 d, 1 w, 2 w, 3 w: 3 days, 1 week, 2 weeks, 3 weeks post-trauma, respectively. Quantification was based on at least 10 fields per section. *P<0.05 vs. normal group.

H&E staining confirmed the presence of osteonecrosis in all sections of traumatized femoral heads; obvious pathological changes were observed as early as 3 days after trauma. For example, the operative medulla on both sides of the epiphyseal plate was found to be loose and greatly diminished, and was worse 1 week after trauma, accompanied by a change of shape or fusion of most medullary cells (Figure 2A, B-a). Hemorrhage was evident and increased for 2 weeks, after which it became limited. Simultaneously, immature fibrous tissue was observed in the medulla under the cartilage. To further examine alterations in the vasculature, microvessels (MVs) were labeled with vWF stain [20], a marker of endothelial cells, and observed by immunohistochemistry (IHC) (Figure 2C, D). MVs were abundant in normal tissues, but decreased rapidly after trauma. Loss of cell nuclei marked by hematoxylin staining, along with non-regular spacing of vascular endothelial cells (VECs), was observed at the end of the study. The three-layer structure of arteries was observed only up to 3 days after trauma, and gradually broke down thereafter. Until 3 weeks after trauma, the MVs marked by CD105, a specific marker of new blood vessels, could still be observed but were limited in number in the injured femoral head (Figure 2E, F).

In injured tissue, concomitant increases in OCs inferred by morphological features and empty lacunae up to week 2 after trauma were observed (Figure 2A, B-b), accompanied by the gradually increased thinning of the trabeculae (Figure 2B-c) and enlargement of the medullary cavities from 1 week after trauma. These findings were consistent with the lack of focal signal intensity at the subchondral bone on radiological examination and may be associated with loss of heavy-loading capacity. Beginning 3 days after trauma, small regions within the trabeculae near the cartilage exhibiting a grey color similar to cartilage appeared, and increased markedly 2 weeks after trauma (Figure 2B-d). Some of these regions stained with eosin, indicating new bone formation. Appositional bone formation was also observed in the metaphysis, but had decreased greatly by week 3 post-trauma. Spindle-shaped osteoblasts (OBs) were readily observed in an orderly arrangement near the epiphyseal plate, although they were reduced in number up to 1 week after trauma and became distant to the trabeculae over time (Figure 2). An obvious and noteworthy concomitant increase in OBs was observed 2 weeks after trauma, especially under the cartilage where OBs were originally sparse.

Both radiological and pathological examination confirmed that a model of early stage traumatic ONFH had been established. Several pathological observations suggested the occurrence of self-tissue repair, including the appearance of revascularization and the increases in immature fibrous tissue in the medulla, grey regions in the trabeculae and OBs under the cartilage.

### Tissue self-repair in the local region after trauma

As tissue self-repair is important for the choice of timing and method of treatment in ONFH, it is worth clarifying its temporal and spatial features. For this purpose, markers of OB regeneration were determined by IHC, including the cell proliferation marker proliferating cell nuclear antigen (PCNA) and the bone formation markers, collagen type I (Col I) and osteocalcin (OCN).

**Figure 3:**
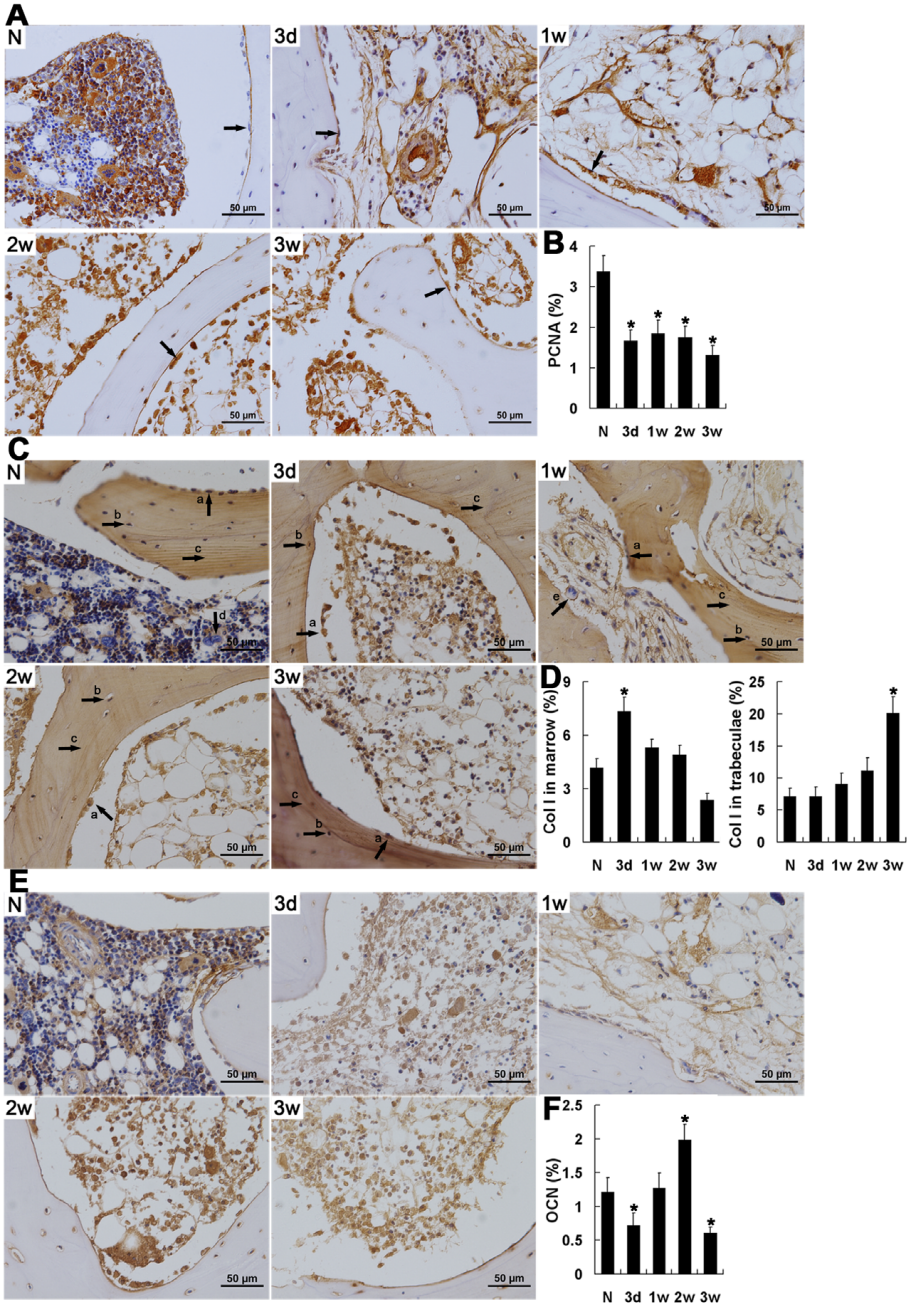
Tissue self-repair after trauma indicated by immunohistochemical staining and semi-quantitative analysis of PCNA, Col I and OCN. (A, C, E) Immunohistochemical assays of the expression of PCNA (A), Col I (C) and OCN (E). (B, D, F) Bar graphs represent their expression density in unit area of bone marrow or unit area of bone trabeculae. (A) OBs; (C) a. OBs, b. osteocytes, c. trabeculae, d. VECs, e. OCs. The expression of PCNA decreased after trauma, and began to be detected in OBs 2 weeks after trauma and was maintained during the following time. Col I increased in the bone marrow for less than 1 week after trauma, and was significantly accumulated in the trabeculae by the end of the study. Both OBs and OCs also expressed increased levels of Col I following trauma. A significant decrease in OCN was detected 3 days after trauma, but it increased significantly 2 weeks later. High levels of OCN were consistently associated with peri-VECs and the fibrous medulla. N: normal group; 3 d, 1 w, 2 w, 3 w: 3 days, 1 week, 1 weeks, 3 weeks post-trauma, respectively. Quantification was based on at least 10 fields per section. *P<0.05 vs. normal group.

High levels of PCNA were detected in normal hematopoietic tissue, but not in osteocytes or OBs. Following trauma, PCNA levels decreased significantly (*P*<0.01) then were maintained at a relatively stable and low level, especially in hematopoietic tissues. In OBs, PCNA expression began to be detected 2 weeks after trauma and was maintained throughout the remaining observation period, although the number of OBs had decreased by the end of observation (Figure 3A, B).

As the most abundant protein in the extracellular matrix of bone and skin, the large diameter fibrils of Col I are responsible for tissue stabilization and regeneration. Col I levels were low in normal medullary cavities, but increased immediately after trauma and then decreased over the remaining observation period. Col I levels, which were low in the OCs of normal tissues, increased markedly as early as 3 days post-trauma and continued to increase for up to 2 weeks, which indicated an increase in OC activity [21] and the presence of bone remodeling [2]. In contrast, Col I was barely detectable in OBs in normal tissue. After trauma, the Col I level was initially low, but it had increased markedly by 2 weeks post-trauma; this increase was concomitant with PCNA expression and suggested the presence of new OBs in the early stage of OB differentiation. Compared with marrow cells, Col I expression in the trabeculae was elevated following trauma, and remained significantly higher than in normal femoral heads during the third week (*P*<0.01), suggesting decreased OB activity and a failure to degrade Col I for matrix mineralization by the end of the observation period. In VECs, it was notable that, instead of the varied expression of Col I seen in normal tissues, there were uniformly high levels of Col I following trauma (Figure 3C, D).

The level of Col I represents a measure of bone tissue status. Similarly, OCN is a specific marker of bone formation during bone turnover [22], and characterizes the initial osteogenic differentiation of MSCs [23]. In normal femoral head sections, expression of OCN was mainly in perivascular vessels and in a subset of osteocytes. However, 3 days after trauma, OCN levels decreased significantly (*P*<0.05), and this was concomitant with the decrease in the number of OBs. Subsequently, OCN levels returned to normal, and then increased to a higher level again 2 weeks after trauma, although they had decreased significantly overall by the end of week 3. In general, the highest levels of OCN expression were consistently observed in perivascular vessels and in fibrous medulla, regions associated with localization of MSCs [24]. In addition, the re-elevation of OCN expression was coordinated with the increase in the number of OBs (Figure 3E, F), suggesting that osteogenic differentiation of MSCs remained active in the injured tissue.

### Efficacy of transplantation of HGF-transgenic MSCs

The above IHC results, including increased PCNA and Col I in OBs and the repeated increase of OCN, suggest that there was a relatively strong tissue self-repair for up to 2 weeks after trauma. Thus, we treated ONFH model animals with transplantation of transgenic MSCs 1 week after trauma with the aim of improving self-repair.

**Figure 4:**
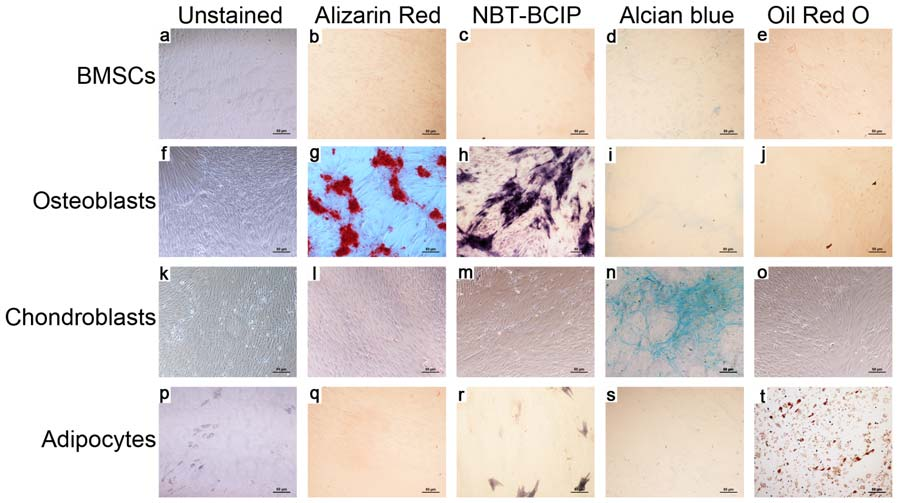
Identification of MSC pluripotent potential. Undifferentiated MSCs (a–e), osteoblasts (f–j), chondroblasts (k–o), and adipocytes (p–t) were left unstained (a, f, k, & p), or stained with Alizarin Red at day 21 (b, g, l, & q), NBT-BCIP at day 14 (c, h, m, & r), Alcian blue at day 27 (d, i, n, & s), or Oil Red O at day 27 (e, j, o, & t). Scale bar = 50 µm.

**Figure 5:**
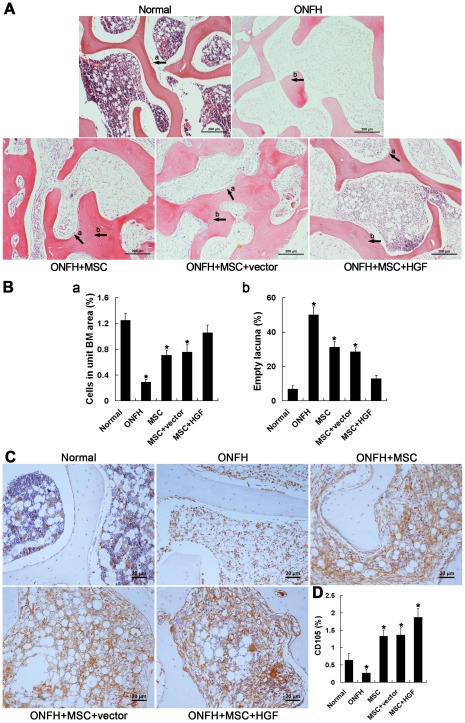
Histopathological examination of treatment efficacy in traumatic ONFH by H&E staining, and immunohistochemical staining and semi-quantitative analysis of CD105. (A) Representative image of a section of femoral head stained with H&E. Scale bar = 200 µm. In the treated femoral head, the number of empty lacunae decreased and hematopoietic tissue partially recovered, accompanied by an increase in the number of OBs, which was most significant in animals that received HGF-transgenic MSCs. a. empty lacuna; b. OBs. (B) Bar graph (left panel) represents the ratio of bone marrow cells to the area of bone marrow. A second bar graph (right panel) represents the ratio of empty lacunae to the area of trabeculae. (C) Immunohistochemical assays of CD105 expression. Scale bar = 20 µm. (D) Bar graphs represent the expression density of CD105 as unit area of bone marrow or unit area of bone trabeculae. After MSC transplantation, the expression of CD105 increased compared with that of untreated ONFH group, indicating the occurrence of revascularization. Quantification was based on at least 10 fields per section. *P<0.05.

Before transplantation, the pluripotency of the MSCs was verified by inducing their differentiation into osteoblasts, chondroblasts, or adipocytes (Figure 4). H&E-stained specimens collected 4 weeks after transplantation showed virtually no hematopoietic tissue, no live osteocytes, and misarranged, thinning trabeculae in the ONFH group that did not receive any treatment. In contrast, in all treated groups, there was an increase in OBs with an orderly arrangement near the trabeculae, and partial recovery of hematopoietic tissue; in addition, the thinning of the trabeculae was attenuated. However, only in the ONFH+MSC+HGF group did the number of empty lacunae decrease and hematopoietic tissue recover significantly compared with the ONFH group (*P*<0.05). Moreover, normal bone marrow including tri-lineage hematopoietic elements was observed locally, which may have resulted, at least in part, from the sequentially transient increase in HGF-mediated p-ERK/Akt signaling described below. Meanwhile, the trabeculae exhibited a more organized arrangement and more new capillaries were observed in animals treated with HGF-transgenic MSCs compared with the other two treatment groups (Figure 5A, B-a, B-b). The expression of CD105 also increased after MSC transplantation, which suggested the occurrence of new MVs. Although angiogenesis did not recover to normal levels, the amounts of MVs marked by CD105 in the ONFH+MSC+HGF group were significantly stronger than the other two treatment groups in addition to the untreated ONFH group (Figure 5C, D).

**Figure 6:**
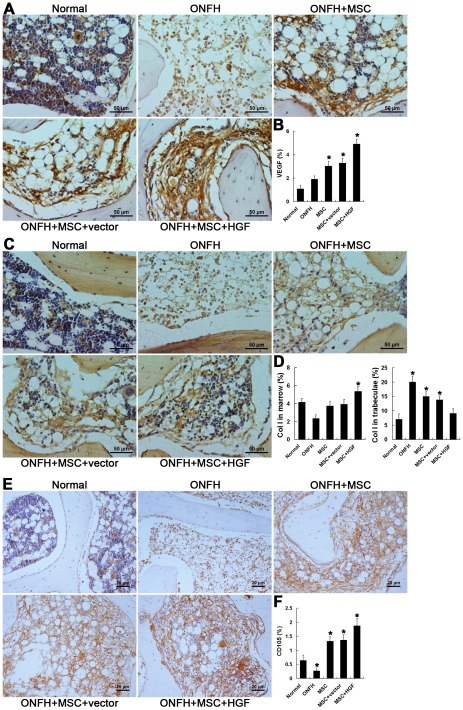
Immunohistochemical staining and semi-quantitative analysis of VEGF and Col I. (A, C) Immunohistochemical assays of the expression of VEGF (A) and Col I (C). (B, D) Bar graphs represent the expression density of VEGF and Col I as unit area of bone marrow or unit area of bone trabeculae. Transplantation of MSCs reversed the decline of VEGF and Col I expression in the marrow, but decreased the accumulation of Col I in the trabeculae. The most significant recovery was seen in the ONFH+MSC+HGF group. Quantification was based on at least 10 fields per section. *P<0.05 vs. normal group.

VEGF is a specific mitogen that acts on endothelial cells. It has multiple functions, including inducing endothelial cell growth, angiogenesis, and vasculogenesis, promoting cell migration and inhibiting apoptosis. VEGF plays important roles in vasculogenesis during the healing of and recovery from bone fracture. It also promotes OB migration through an interaction with the flt-1 receptor, which is highly expressed on the surface of OBs. Thus, elevated VEGF expression after trauma will contribute to healing. In the ONFH model, expression of VEGF after trauma greatly decreased compared with the normal group. However, VEGF levels were markedly increased after MSC transplantation. The greatest levels of expression were observed in animals treated with HGF-transgenic MSCs, which also exhibited the greatest promotion of vasculogenesis (Figure 6A, B).

Col I in medullary cavities also increased gradually after treatment for 2 weeks and was higher in animals in the ONFH+MSC+HGF group than in the other two treatment groups (*P*<0.05) (Figure 6C, D-a). In the trabeculae, treatment by transplantation of HGF-transgenic MSCs decreased, but did not completely inhibit, accumulation of Col I. The most significant decrease of Col I in trabeculae was detected in the ONFH+MSC+HGF group, in which the Col I level increased slightly but was not significantly higher than that in the normal group (Figure 6 C, D-b). These results suggest that animals in the ONFH+MSC+HGF group had the strongest activity of OBs, which promote new bone formation.

### HGF-induced sequential activation of ERK1/2 and Akt contributed to the efficacy of HGF-transgenic MSC transplantation

Our previous work showed that HGF levels in the femoral head region in hormone-induced ONFH changed after transplantation with HGF-transgenic MSCs which promotes the function of MSCs in tissue repair. At the beginning of treatment, high levels of HGF enhance MSC proliferation by strongly activating ERK1/2 signaling; a gradually decreasing HGF level then promotes MSC osteogenic differentiation through preferential activation of the Akt pathway [16].

**Figure 7:**
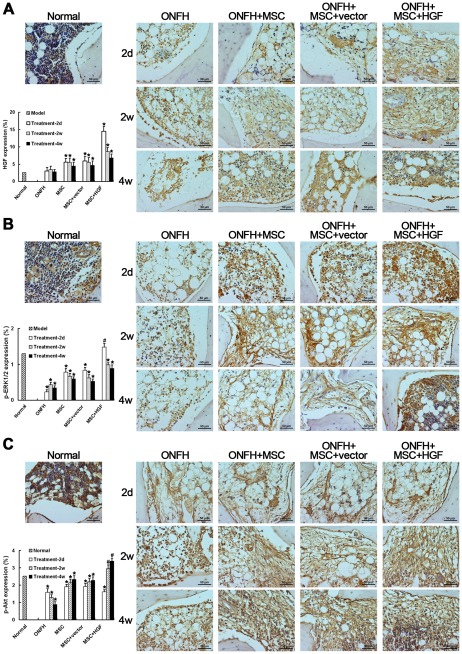
Immunohistochemical detection and semi-quantitative analysis of HGF expression (A), phosphorylation of ERK1/2 (p-ERK1/2) (B) and Akt (p-Akt) (C). There was a little increase in HGF after trauma. After the transplantation of MSCs, the HGF level increased significantly at as early as 2 days, which was concomitant with increased p-ERK1/2. The HGF level decreased gradually for 2 weeks after transplantation, followed by a significant increase in Akt activation. The effects were most marked in the animals treated with HGF-transgenic MSCs. ^*^ *P*<0.05, compared with the normal group. ^#^ *P*<0.05, compared with the non-infected MSC-treated group. Scale bar = 50 µm.

To investigate whether a similar mechanism exists in the traumatic ONFH model, we explored the mechanism by which HGF-transgenic MSC transplantation achieves its therapeutic effect. Firstly, we measured HGF expression levels in all treated tissues. Unexpectedly, HGF protein expression increased slightly in the traumatic ONFH model group before eventually declining, distinguishing the present model from hormone-induced ONFH. Significant increases in HGF expression were observed in all of the treated animals as early as 2 days after transplantation and decreased approximately 2 weeks later (Figure 7A). The greatest levels of HGF were observed in animals treated with HGF-transgenic MSCs. Unlike HGF, which increased after trauma, p-ERK1/2 levels, which were high in the normal group, were greatly decreased in the ONFH group. Transplantation of MSCs greatly increased activation of the ERK1/2 pathway; the highest p-ERK1/2 levels were still seen in the animals treated with HGF-transgenic MSCs, concomitant with a significant elevation of HGF expression (Figure 7B). Although lower than in the normal group, Akt pathway activation was still extensive in the ONFH group, and was elevated after MSC transplantation. However, a significant increase in Akt pathway activation was observed only in the ONFH+MSCs+HGF group after 2 weeks (Figure 7C), accompanied by a decline in HGF expression in the local region. These observations demonstrate that the transplantation of HGF-transgenic MSCs contributed to recovery from traumatic ONFH through a mechanism similar to that seen with treatment of hormone-induced ONFH [16].

## Discussion

In this study, a model of early stage traumatic ONFH representing clinical ARCO phase I or II ONFH was established in rabbits and confirmed by CT and MR imaging and pathological examination. Based on the differing distributions of necrotic lesions on the two flanks of the epiphyseal plate in this model and the preserved cartilage, which are consistent with human ONFH, this model can be regarded as a mimic of early stage clinical ONFH. Based on the apparent occurrence of self-repair after trauma, we selected the best time for treatment of the model with the transplantation of HGF-transgenic MSCs. Local inspection of the treated tissues confirmed the efficacy of this therapeutic method and the feasibility of assessing its efficacy in this traumatic ONFH model.

Complicated cellular interactions in the injured femoral head contribute to the development of ONFH. According to the histological definition of osteonecrosis, including empty lacunae in the osseous matrix and necrosis of marrow elements [25], [26], osteonecrosis occurred within 3 days after trauma. During the progression of traumatic ONFH, there was obvious evidence of tissue self-repair. For example, pathological inspection with H&E staining showed the presence of local bone formation as well as the reoccurrence of OBs, which was especially obvious under the cartilage. Furthermore, expression of CD105, a specific marker of new blood vessels [27], [28], [29], increased around the MVs until 3 weeks after trauma, which suggested the emergence of revascularization. In addition, although PCNA levels decreased following traumatic surgery, the increase in OBs from the second week indicated their proliferation during the early stage of OB differentiation. At the same time, as a marker of initiation of osteogenic differentiation, which is elevated in the early stage of bone synthesis after trauma [23], expression of OCN also reached its highest level 2 weeks after trauma. All of these observations indicate that there was ongoing tissue self-repair in the injured femoral head until for 2 weeks after trauma. Our assessment of tissue self-repair was based mainly on the expression of marker molecules. Nonetheless, as the trauma operation did not injure the medullary cavity, the residual MSCs, the intact cartilage, the epiphyseal plate, and the connective tissue around or infiltrating into the femoral head, may also play roles in this process. However, a continual decrease in the MVs marked by vWF indicated that there was an increased rate of apoptosis of endothelial cells after trauma. Additionally, the continued increase in OCs and empty lacunae as well as thinning of the trabeculae suggest that there was an imbalance between osteogenesis and osteoclastic absorption, which may account for the bone loss. Due to the complete disruption of vascularization, self-repair could not keep pace with the ongoing tissue necrosis and became abortive, resulting in irreversible progression of the disease.

It is proposed that a treatment that promotes revascularization and bone tissue repair before the peak level of self-repair at approximately 2 weeks after trauma would improve the efficacy of tissue self-repair, impede the progression of the disease, and potentially rescue a necrotic femoral head. The course of self-tissue repair provided an excellent time point to intervene in the development of the disease and suggested that pluripotent MSCs could play important roles in this process. Accordingly, we treated the traumatic ONFH model animals with transplanted MSCs transfected with HGF early after trauma before the reparative phase was histologically observed. One week after trauma, the injured tissue had recovered more with transplantation of HGF-transgenic MSCs than with uninfected or Ad-vector-infected MSCs. Changes in HGF expression were also observed; HGF levels increased immediately after transplantation and declined approximately 2 weeks later. This pattern of HGF expression was the same as that seen *in vitro* with Ad-HGF-transfected MSCs and *in vivo* treatment of hormone-induced ONFH [16]. Our previous work demonstrated that the elevation of HGF promotes MSC proliferation to meet the cell's requirement for tissue reconstruction through the activation of the ERK1/2 signaling pathway. The subsequent decrease in HGF promotes MSC differentiation by activating the Akt signaling pathway in the osteogenic microenvironment, favoring re-establishment of bone tissue, as demonstrated by increased numbers of OBs. These results suggest that changes in HGF concentration enhance the ability of MSCs to contribute to femoral head tissue repair in ONFH. Similar phenomena were also observed in the untreated animals up to 2 weeks after trauma, but they were much less extensive, suggesting that coordination between the therapy and self-repair promoted recovery in the treated animals. The activation of the ERK1/2 and Akt pathways during the treatment of traumatic ONFH was also similar to that observed in the treatment of hormone-induced ONFH with the transplantation of HGF-transgenic MSCs [16]; that is, ERK1/2 activation increased simultaneously with increased HGF expression after transplantation, but decreased about 2 weeks later. In contrast, there was less activation of the Akt pathway after MSC transplantation than 2 weeks later, and these changes in Akt activations were accompanied by changes in HGF levels. These observations suggest that, in traumatic ONFH, HGF affects the activity of MSCs through a mechanism similar to that seen in hormone-induced ONFH. That is, high concentrations of HGF enhance activation of the ERK1/2 signaling pathway and inhibit that of Akt, which promotes MSC proliferation but suppresses osteogenic differentiation. However, low concentrations of HGF can activate Akt and promote osteogenic differentiation [16]. It should be noted that the changes in Akt activation observed in traumatic ONFH were not as significant as in hormone-induced ONFH. The reason may be the presence of tissue self-repair. It was observed that, although declining, expression of Akt was still higher in our animals than in hormone-induced ONFH [16]. For this reason, the increase of Akt activation resulting from transgenic HGF was limited.

The self-repair observed in the traumatic ONFH model suggested that significant MSC activity was present in the injured tissue. Strong signals persisted around VECs until the end of the observation period, consistent with the presence of MSCs [24]. Such phenomena were not observed in hormone-induced ONFH, suggesting that MSCs may have been inactivated after hormone treatment. This possibility is consistent with the report of Weinstein *et al*. [30], and such findings suggest that MSC activity is important for recovery from ONFH. In traumatic ONFH, the use of MSC transplantation to coordinate tissue self-repair could improve the effects of the latter, and the pro-osteogenic function on MSCs of HGF could further increase the efficacy of the treatment. Besides the effects on MSCs, previous studies have reported that HGF can induce secretion of VEGF through activation of its receptor c-Met, which is followed by the activation of both the ERK1/2 and Akt signaling pathways [31], . In bone marrow, c-Met is extensively expressed on MSCs and endothelial cells as well as other cell types. The transplantation of HGF-transgenic MSCs is therefore a promising potential therapy for traumatic ONFH. In the future, further investigations of the etiology of ONFH and the effects of the therapeutic transplantation of HGF-transgenic MSCs will provide a deeper understanding of the progression of ONFH after treatment and will be of assistance in designing more effective therapeutic regimens.

### Conclusions

In this study, a rabbit model of early stage traumatic ONFH was established. Based on radiological inspection and the distribution and features of necrotic lesions, this model can be considered to represent clinical ARCO phase I or II ONFH and to be suitable for pathophysiological studies and the assessment of therapeutic regimens. Histological findings and the expression profiles of specific molecules indicate that self-repair was initiated in the femoral head as early as 3 days after trauma and peaked at 2 weeks. The use of this therapeutic window will be critical for designing therapeutic regimens. Transplantation of HGF-transgenic MSCs was performed 1 week after trauma and contributed greatly to the recovery of the injured femoral head. These results provide valuable insights into the development of effective treatment strategies for ONFH.
